# Astrocyte Senescence and Metabolic Changes in Response to HIV Antiretroviral Therapy Drugs

**DOI:** 10.3389/fnagi.2017.00281

**Published:** 2017-08-29

**Authors:** Justin Cohen, Luca D’Agostino, Joel Wilson, Ferit Tuzer, Claudio Torres

**Affiliations:** Department of Pathology and Laboratory Medicine, Drexel University College of Medicine, Philadelphia PA, United States

**Keywords:** cellular senescence, highly active antiretroviral therapy, HIV, astrocytes, glycolysis, HIV-associated neurocognitive disorders

## Abstract

With the advent of highly active antiretroviral therapy (HAART) survival rates among patients infected by HIV have increased. However, even though survival has increased HIV-associated neurocognitive disorders (HAND) still persist, suggesting that HAART-drugs may play a role in the neurocognitive impairment observed in HIV-infected patients. Given previous data demonstrating that astrocyte senescence plays a role in neurocognitive disorders such as Alzheimer’s disease (AD), we examined the role of HAART on markers of senescence in primary cultures of human astrocytes (HAs). Our results indicate HAART treatment induces cell cycle arrest, senescence-associated beta-galactosidase, and the cell cycle inhibitor p21. Highly active antiretroviral therapy treatment is also associated with the induction of reactive oxygen species and upregulation of mitochondrial oxygen consumption. These changes in mitochondria correlate with increased glycolysis in HAART drug treated astrocytes. Taken together these results indicate that HAART drugs induce the senescence program in HAs, which is associated with oxidative and metabolic changes that could play a role in the development of HAND.

## Introduction

With the advent of highly active antiretroviral therapy (HAART), HIV infection has transitioned from an acute, terminal illness to a chronic but manageable condition (Bhatia et al., 2012). The HIV-infected population is consequently aging, and it had been projected that by 2015 more than 50% of the HIV-infected population in the United States would be 50 years of age and older. While this is undoubtedly a major success, aging is a significant risk factor for disease (Niccoli and Partridge, 2012) and HIV patients experience a variety of age-related complications, suggesting premature aging (Capeau, 2011). One such complication is a series of neurological problems collectively known as HIV-associated neurocognitive disorders (HAND) (Heaton et al., 2010). HAND can be categorized with increasing severity from asymptomatic neurocognitive impairment, mild neurocognitive disorder, and HIV-associated dementia. While the prevalence of HIV-associated dementia in the post-HAART era has decreased in HIV-infected patients, asymptomatic neurocognitive impairment and mild neurocognitive disorder have increased (Heaton et al., 2010). The persistence of neurological problems in HIV-infected patients remains a major public health issue and the identification of mechanisms involved may lead to potential treatments.

While beneficial in their suppression of HIV, HAART drugs have a multitude of side effects including myopathy, hepatotoxicity, hypersensitivity reactions, lipodystrophy, and insulin resistance (Feeney and Mallon, 2010). *In vitro*, there has been evidence of HAART drugs inducing ER stress (Sato et al., 2012), unfolded protein response (Zhou et al., 2005), and changes to cellular metabolism (Arend et al., 2013). These side effects suggest that cells may undergo a great deal of stress in response to HAART drugs. One possible way that cells can respond to stress is to undergo cellular senescence.

Cellular senescence is an age-related phenotype originally discovered to occur *in vitro* after extensive cell passaging, and is associated with the telomere attrition that occurs with successive rounds of DNA replication (Bodnar et al., 1998). Senescence also occurs prematurely in response to other mediators. Oncogene-induced senescence can occur via the activation of tumorigenic signals such as telomere dysfunction (Suram et al., 2012) and oncogenic RAS (Serrano et al., 1997). Stress-induced premature senescence can occur in response to cytotoxic stimuli such as proteasome inhibition and oxidative stress (Chen et al., 1995; Bitto et al., 2010). Several classes of HAART drugs including nucleoside reverse transcriptase inhibitors and protease inhibitors have been shown to cause stress-induced premature senescence (Caron et al., 2008; Lefevre et al., 2010; Hernandez-Vallejo et al., 2013; Afonso et al., 2015), suggesting that HIV patients may be experiencing cellular senescence. Evidence for cellular senescence during HIV comes from a previous study showing increased senescent CD8^+^ T-cells isolated from HIV patients (Chou et al., 2013). Regardless of the inducer, there are several phenotypes and biomarkers generally shared among senescent cells. These include cell cycle arrest, increased senescence-associated beta-galactosidase (SA β-gal) activity, expression of the cell cycle inhibitors p16 and p21, mitochondrial dysfunction, and the secretion of pro-inflammatory cytokines and proteases known as the senescence-associated secretory phenotype (SASP) (Rodier and Campisi, 2011). The pro-inflammatory environment created by the SASP has major implications for age-related decline in tissues and may contribute the chronic inflammation observed in the central nervous system (CNS) in neurological diseases such as Parkinson’s and AD (Jabbari Azad et al., 2014; Yan et al., 2014) and HAND.

Senescence in the CNS is an emergent concept and few studies have examined its role as a contributor to neurodegenerative disease. Astrocytes are the most abundant cells in the brain and are involved in a variety of functions to maintain CNS homeostasis such as CNS metabolism, blood brain barrier maintenance, and ion regulation (Stobart and Anderson, 2013). Due to their numerous functions in the CNS, disruption of their physiological functions due to senescence could be a major contributor to neurological disease. Our recent work demonstrates a decrease in astrocyte-enriched genes during senescence, indicating a loss in their differentiated function (Crowe et al., 2016). This could impact brain physiology in Alzheimer’s patients where we have previously reported a significant increase in the population of senescent astrocytes (Bhat et al., 2012). In the present study, we evaluated the role of HAART drug exposure on astrocyte senescence. Human astrocytes (HAs) treated with a clinically relevant combination of nucleotide reverse transcriptase inhibitors and protease inhibitors underwent cellular senescence with expression of p16, p21, SA β-gal, and pro-inflammatory cytokines. The process was accompanied with increased oxidative stress, mitochondrial oxygen consumption, and changes in glucose metabolism with increased glucose uptake and upregulation in glycolytic intermediates. To our knowledge, our findings are the first to demonstrate HAART drug-induced senescence in a CNS cell type, which may have implications for HAND.

## Materials and Methods

### Cell Culture and Drug Treatments

Human astrocytes were cultured at 37°C, 5% CO_2_ in astrocyte medium supplemented with 2% fetal bovine serum, growth supplement, and penicillin/streptomycin all obtained from ScienCell Research Laboratories (Carlsbad, CA, United States). Cells were cultured until they reached ∼80% confluence before passaging. At each passage, astrocytes were trypsinized, counted, and the cumulative population doubling level (CPDL) was calculated as we have previously described (Bitto et al., 2010). Cells were treated every 2–3 days for up to a week with either 0.3% DMSO as a vehicle control or the HAART drug combinations of abacavir (ABC) 10 μM and lamivudine (3TC) 5 μM or ABC, 3TC, and ritonavir (RTV) 1 μM. For the long-term experiments, cells were treated for up to 4 weeks with either 0.2% dimethyl sulfoxide (DMSO) as a vehicle control or the combinations of ABC 3 μM, 3TC 1.9 μM, atazanavir (ATV) 50 nM, and RTV 100 nM; or tenofovir (TDF) 100 nM, emtricitabine (FTC) 1.2 μM, ATV, and RTV; or TDF, FTC, and efavirenz (EFV) 125 nM. All HAART drugs were provided by the NIH AIDS Reagent Program.

#### Senescence-Associated β-Galactosidase Activity Assay

Senescence-associated beta-galactosidase staining was performed as previously described (Dimri et al., 1995). Briefly, following exposure to the HAART drug combinations or DMSO, astrocytes were fixed in 2% formaldehyde/0.2% glutaraldehyde for 3 min and stained for SA β-gal activity overnight. The cells were counted and positive (blue) cells were expressed as a percentage of the total. At least 200 cells were counted.

#### Immunoblotting

Following indicated treatment times, HAs were lysed in radioimmunoprecipitation assay (RIPA) buffer. Western blot analysis was performed under standard conditions using 15 μg of total cell proteins. Membranes were probed for antibodies against p16 [sc-56330 (**JC8**), monoclonal; BD Biosciences, San Jose, CA, United States]; p21 [sc-756 (**H-164**), polyclonal; Santa Cruz Biotechnology, Santa Cruz, CA, United States]; phosphorylated (9211) and total p38 (9212) both polyclonal (Cell Signaling Technology, Danvers, MA, United States); phosphorylated (3033) and total p65 (3034) both polyclonal (Cell Signaling Technology, Danvers, MA, United States); Oxphos cocktail of mitochondrial ETC complexes I [ab110242 (**20E9DH10C12**)], II [ab14714 (**21A11AE7**)], III [ab14745 (**13G12AF12BB11**)], IV [ab110258 (**12C4F12**)], and V [ab14748 (**15H4C4**)] (monoclonal; Abcam, Cambridge, MA, United States); β-actin [A00702 **(2D1D10)**, monoclonal; Genscript, Piscataway, NJ, United States); and β-tubulin [sc-9104 (**H-235**), polyclonal; Santa Cruz Biotechnology, Santa Cruz, CA, United States). Clone numbers are in bold.

### Total Cellular ROS, Mitochondrial ROS, Mitochondrial Membrane Potential, Mitochondrial Mass, and Glucose Uptake Assessments

Determination of total cellular reactive oxygen species (ROS), mitochondrial ROS, mitochondrial membrane potential, mitochondrial mass, and glucose uptake assessments was made using flow cytometry as previously described (Nacarelli et al., 2016). Total cellular ROS was detected by incubating cells with 10 μM 2′7′-dichlorofluorescein diacetate (DCF-DA; Sigma-Aldrich, St. Louis, MO, United States) in 1% fetal bovine serum-supplemented MEM and washing twice with Krebs Ringer phosphate glucose buffer (145 mM NaCl, 5.7 mM NaH_2_PO_4_, 4.86 mM KCl, 0.54 mM CaCl_2_, 1.22 mM MgSO_4_, and 5.5 mM glucose) following incubation. All other compounds were washed with PBS. Mitochondrial superoxide levels were assayed by incubating the cells with 5 μM Mito-Sox Red (Molecular Probes, Waltham, MA, United States). Mitochondrial membrane potential was detected by incubating cells with 25 nM tetramethylrhodamine (TMRE) (Molecular Probes, Waltham, MA, United States). Mitochondrial mass was evaluated by incubating the cells with 100 nM Mito-tracker Green FM (Molecular Probes, Waltham, MA, United States). The glucose analog 2-NBDG uptake was detected by incubating the cells with 10 μM of 2-NBDG (Molecular Probes, Waltham, MA, United States). For the previous analyses, incubation was performed at 37°C in 5% CO_2_ for 30 min except 2-NBDG which required 90 min. Cells were collected in 0.25% trypsin-EDTA with complete medium. Cells were analyzed by flow cytometry using a Guava EasyCyte Mini and the Guava Express Plus program (Guava Technologies, Hayward, CA, United States). Acquisitions involved 5000 events.

### Analysis of Inflammatory Factors Secreted by Astrocytes

Following the end of the treatment period, HAs were incubated with serum-free MCDB105 media. After a 24-h incubation period, media were collected and cells were trypsinized and counted to determine the cell number for normalization. Human Cytokine Array C5 (RayBiotech, Norcross, GA, United States) was used to evaluate secreted inflammatory factors in the conditioned media according to the company’s protocol. The intensity of the signal on the array membranes was quantified by densitometry using ImageJ software and normalized to cell number. The HAART drug treated values were then set as relative to control values. Samples that had no change in expression due to levels being undetectable from background were set to 1. Interleukin-6 (IL-6) detection was performed via the Human IL-6 Quantikine ELISA kit (R&D Systems, Minneapolis, MN, United States) according to the product manual using conditioned media as described above. Absorbance was measured at 450 nm.

### Oxygen Consumption Measurements

Oxygen consumption was determined by using a Seahorse XF24 Bioanalyzer (Seahorse Bioscience, North Billerica, MA, United States) and the XF Cell Mito Stress Test Kit as previously reported (Nacarelli et al., 2016). Cells were seeded after treatment at 25,000 cells per well. The Bioanalyzer was pre-loaded with oligomycin, carbonilcyanide *p*-triflouromethoxyphenylhydrazone (FCCP), and rotenone/antimycin A prior to measurement. Oxygen consumption was measured in triplicate before and after consecutive addition of oligomycin, FCCP, and rotenone/antimycin A. Respiration rates and proton leak were assessed as previously described (Hill et al., 2012) based upon oxygen consumption rate measurements. Basal respiration represents the initial oxygen consumption rate, while maximal respiration signifies oxygen consumption after FCCP addition. ATP-linked respiration denotes the oligomycin-sensitive oxygen change to basal oxygen consumption rate. Proton leak corresponds to the oligomycin-insensitive oxygen consumption rate. Non-mitochondrial sources of oxygen consumption were subtracted by normalizing to the rotenone/antimycin A-insensitive oxygen consumption rate. Acidification was based on the extracellular acidification rate. Data were normalized to cell number.

### Metabolite Measurements

Metabolite measurements were performed by Human Metabolome Technologies America Inc. (Boston, MA, Unites States) using their C-Scope analysis. Samples were collected according to their protocol and sent overnight on dry ice, after which capillary electrophoresis mass spectrometry was performed. Quantifications were performed by hierarchical cluster analysis (HCA) and principal component analysis (PCA) by Human Metabolome Technologies America Inc.’s statistical software.

### Statistical Analysis

Data were either compared using a two-tailed Student’s *t*-test when two groups were involved or one-way analysis of variance (ANOVA) followed by Bonferroni correction when three groups were analyzed. Normality was determined using a Shapiro–Wilk Test with the caveat that low sample sizes can reduce the accuracy of normality tests. Means were derived from at least three independent experiments. Error bars on graphs reflect standard deviation (SD). Statistical significance was considered at *p* < 0.05.

## Results

### HAART Drugs Induce Senescence Program and Inflammatory Response in Human Astrocytes

**FIGURE 1:**
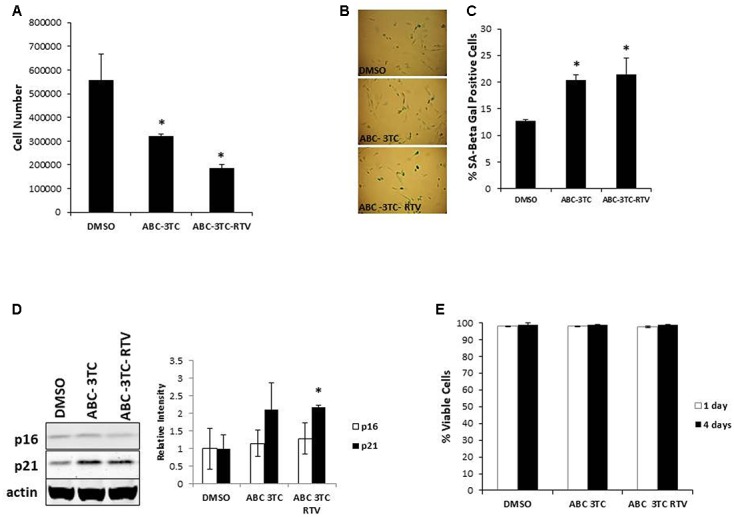
Expression of senescence markers in HAART drug treated human astrocytes (HAs). Human astrocytes were treated with the following HAART concentrations: abacavir (ABC) 10 μM, lamivudine (3TC) 5 μM, and ritonavir (RTV) 1 μM for 7 days **(A–D)** or 4 days **(E)** in complete astrocyte media. **(A)** Cell proliferation. **(B)** Representative SA β-gal images displayed at 20× of HAs stained for SA β-gal 1 week after HAART treatment. **(C)** Quantification of B. **(D)** *Left*—representative Western blot illustrating protein levels of senescence markers p16 and p21. *Right*—quantification of the blots. **(E)** Viability, astrocytes were incubated with Guava Viacount reagent for 5 min prior to detection by flow cytometry. ^∗^*p*-value < 0.05, *n* = 3, error bars are SD.

HIV-infected patients do not take individual antiretroviral drugs but rather they are put on a regimen that includes several different drug classes. We therefore evaluated whether a clinically relevant combination of HAART drugs could affect physiology of HAs. Cells were chronically treated with the either the nucleotide reverse transcriptase inhibitors (NRTIs) ABC and 3TC alone or in combination with the protease inhibitor RTV. Determination of the effects on cell proliferation indicates that after 1 week of treatment these drugs induce a reduction in the cell number compared to vehicle control **(Figure 1A)** which paralleled an increase in SA β-gal activity **(Figures 1B,C)**. Protein levels of the senescence marker p21 but not p16 was significantly increased in the ABC–3TC–RTV combination, suggesting that the pathway may be p21-dependent **(Figure 1D)**. However, the ABC–3TC combination did not show a significant increase in either p16 or p21. In order to rule out the induction of apoptosis, cell viability was measured 1 and 4 days after treatment. No reduction in cell viability was observed compared to the vehicle **(Figure 1E)**. These results suggest that the ABC–3TC and ABC–3TC–RTV induced reduction in HA growth is the result of induction of the senescence program.

In addition, we examined the effect of several other clinically relevant HAART regimens on HA senescence at doses near what is found in the CNS (de Almeida et al., 2006). Human astrocytes were treated with the PIs ATV and RTV with an NRTI backbone of ABC and 3TC; ATV and RTV with an NRTI backbone of TDF and FTC; or TDF and FTC with the NNRTI EFV. Studies of the effects of these drugs on the replicative capacity indicate that compared to DMSO vehicle, there was a statistically significant decrease in population doublings by 11 days of treatment, which broadened further after 35 days **(Supplementary Figure S1A)**. The effects of these drugs were observed even at lower concentrations and the increase in SA β-gal positive cells was significant starting at 1 week, which trended upward at 3 weeks **(Supplementary Figures S1B,C)**. The exception to this trend was the TDF–FTC–EFV combination, which did not increase any further than its 1 week value. Interestingly, at these lower concentrations, ABC–3TC–ATV–RTV does not induce expression of p16 and p21 after 1 week **(Supplementary Figure S1D)**, even though an increase in SA β-gal was observed. This seems to match the minimal impact on cell growth at 1 week, and suggests that changes in the activity of SA β-gal may occur before other markers of senescence.

**FIGURE 2:**
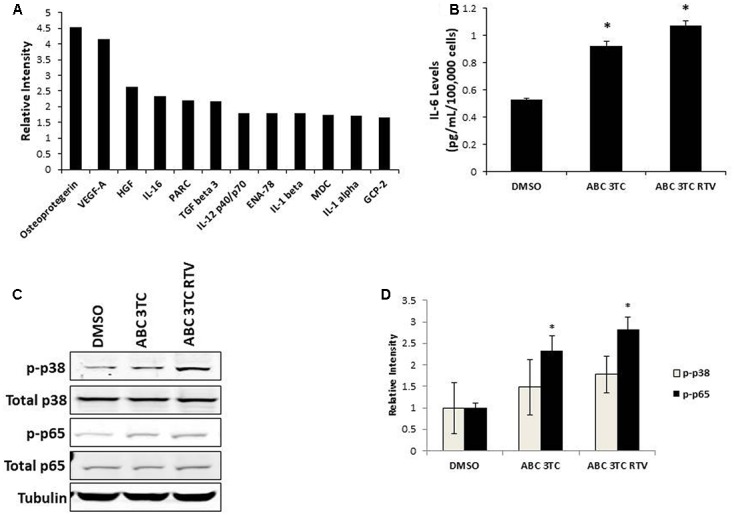
Secretory profile of HAART drug treated HAs. Human astrocytes were treated with the following HAART concentrations: ABC 10 μM, 3TC 5 μM, and RTV 1 μM for 1 week. **(A)** Senescence-associated secretory phenotype (SASP) analysis. Astrocytes were subjected to a 24-h incubation in MCBD105 media to generate conditioned media (CM). The secretory profile was detected by incubating CM on a cytokine membrane array and normalized to cell number. Values are relative to a DMSO control. **(B)** IL-6 quantitation. CM was collected as in **(A)** and IL-6 was quantitated by ELISA. **(C)** Representative Western blot illustrating phosphorylated protein levels of inflammatory mediators p38 and p65. Total p38, total p65, and tubulin were used as a loading control. **(D)** Quantification of **(C)**. ^∗^*p*-value < 0.05, *n* = 3, error bars are SD.

In order to examine the effects of HAART on the SASP, we characterized the secretory pattern of ABC–3TC–RTV-treated HAs using an antibody array. A total of 68 cytokines were analyzed and their relative levels of expression compared to control untreated are shown in **Table 1**. Treatment modulated the secretion of a variety of inflammatory molecules including TGF-β3, IL-1α, and IL-1β **(Figure 2A)**. Further validation these cytokines could be particularly interesting as they have been shown to induce senescence in neighboring cells through a process called paracrine senescence (Acosta et al., 2013). Little change was found in IL-6 using the cytokine array. This may be due to a sensitivity issue of the membrane-based analysis; therefore, we also examined IL-6 via ELISA. One week of treatment with the ABC–3TC and ABC–3TC–RTV HAART drug combination induced a significant, nearly twofold increase in IL-6 release **(Figure 2B)**. The CNS-based ABC–3TC–ATV–RTV combination was also able to induce IL-6 secretion over time in HAs, with a nearly threefold increase after 4 weeks of treatment **(Supplementary Figure S1E)**. Importantly, the pro-inflammatory transcription factor p65 (NF-κB), which has been shown to mechanistically induce senescence (Freund et al., 2011; Tilstra et al., 2012), is activated in response to both ABC–3TC and ABC–3TC–RTV treatment **(Figures 2C,D)**. Another pro-inflammatory mediator p38 while trending upward did not reach statistical significance **(Figures 2C,D)**.

**Table 1:** Senescence-associated secretory phenotype of ABC–3TC–RTV-treated human astrocytes.

Name	ABC–3TC–RTV	Name continued	ABC–3TC–RTV continued
Angiogenin	0.53	IL-8	0.75
BDNF	0.59	IP-10	0.77
EGF	0.86	Leptin	1.00
Eotaxin-1	0.81	LIF	0.98
Eotaxin-2	0.89	Light	1.05
Eotaxin-3	0.83	MCP-1	0.73
FGF-4	0.75	MCP-4	1.00
FGF-6	0.82	M-CSF	0.82
FGF-9	1.27	MDC	1.74
Fractalkine	1.03	MIF	1.31
GCP-2	1.66	MIG	1.00
G-CSF	1.00	MIP-1 beta	1.16
GDNF	1.06	NAP-2	0.93
GM-CSF	1.00	NT-3	0.60
GRO	0.73	NT-4	0.58
GRO alpha	1.00	Oncostatin M	0.84
HGF	2.64	Osteopontin	0.39
I-309	1.00	Osteoprotegerin	4.53
IGF-1	0.69	PARC	2.19
IGFBP-1	1.08	PDGF-BB	1.00
IGFBP-2	0.86	PLGF	1.16
IGFBP-3	0.56	RANTES	1.04
IGFBP-4	1.39	TARC	1.00
IL-1 alpha	1.71	TGF beta 1	1.00
IL-1 beta	1.78	TGF beta 2	0.89
IL-10	1.58	TGF beta 3	2.16
IL-12 p40/p70	1.80	Thrombopoietin	1.00
IL-13	1.00	TIMP-1	0.89
IL-16	2.34	TIMP-2	0.76
IL-3	1.17	TNF alpha	1.18
IL-4	1.00	TNF beta	0.46
IL-5	1.00	VEGF-A	4.16
IL-6	1.00	CCL23	1.08
IL-7	1.00	ENA-78	1.79

*Cytokines were measured by an antibody array and values are expressed as relative to DMSO control*.

### HAART Drugs Induce Oxidative Stress

**FIGURE 3:**
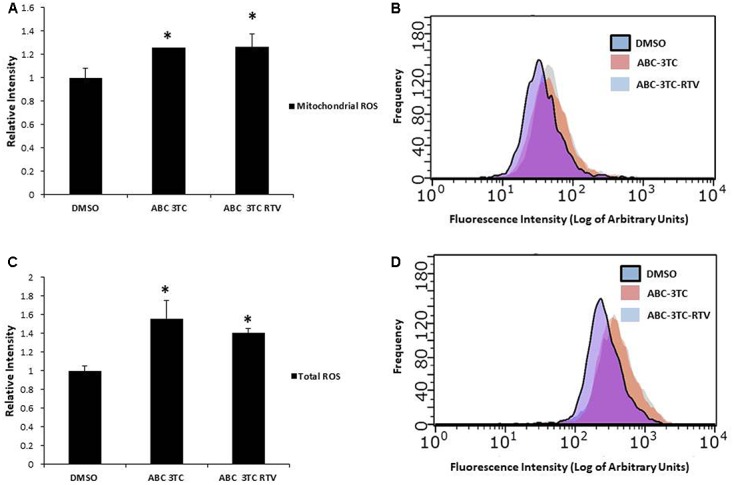
HAART drugs induce total and mitochondrial ROS in HAs. Human astrocytes were treated for 1 week with ABC 10 μM, 3TC 5 μM, and RTV 1 μM before assaying. **(A)** Mitochondrial ROS was measured by incubation with MitoSox for 30 min followed by quantification on a flow cytometer. Bar graphs show mean intensity. **(B)** Representative histogram of data from **(A)**. **(C)** Total ROS was measured by incubation with DCF-DA for 30 min followed by quantification on a flow cytometer. Bar graphs show mean intensity. **(D)** Representative histogram of data from **(C)**. ^∗^*p*-value < 0.05, *n* = 5, error bars are SD.

Accumulation of ROS can induce oxidative stress and contribute to premature senescence (Chen et al., 1995). The main source of ROS is the mitochondrial-specific superoxide anion, which can be converted to other forms of ROS to cause oxidative damage (Wang et al., 2013). We examined the effects of HAART drug combinations on mitochondrial ROS production in HAs, and we observed that both ABC–3TC and the ABC–3TC–RTV combinations significantly increased mitochondrial ROS **(Figures 3A,B)**. Interestingly, the lower dose combination of ABC–3TC–ATV–RTV at 4 weeks of treatment was able to reach a similar level of mitochondrial ROS **(Supplementary Figure S2A)**. Due to this increase in mitochondrial ROS, we examined if there was a corresponding change in total cellular ROS. With 1 week treatment of ABC–3TC and ABC–3TC–RTV, total ROS significantly increases **(Figures 3C,D)**. A similar increase was seen after prolonged treatment with the lower dose ABC–3TC–ATV–RTV combination **(Supplementary Figure S2B)**. These results indicate that the HAART drugs induce oxidative stress in HAs.

### HAART Drugs Impact Mitochondrial Respiration

**FIGURE 4:**
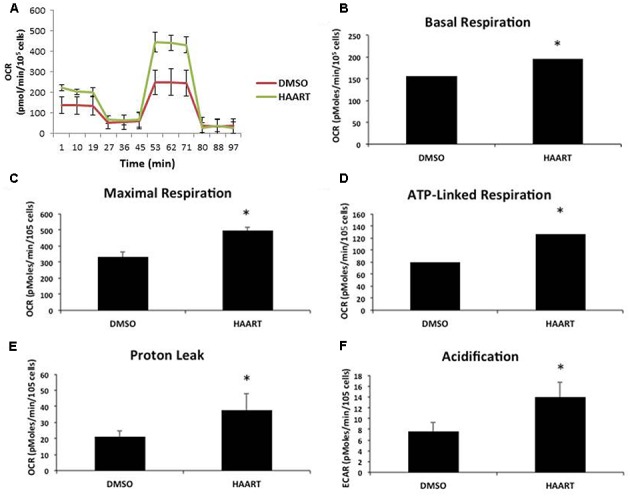
HAART drugs induce mitochondrial respiration. Human astrocytes were treated with the following HAART drug concentrations: ABC 10 μM, 3TC 5 μM, and RTV 1 μM for 1 week before assaying. Oxygen consumption measurements were taken after HAART treatment on a Seahorse XF24 Bioanalyzer using the XF Cell Mito Stress Test Kit to acquire the oxygen consumption rate. **(A)** Representative Seahorse recording. **(B–E)** Bar graphs of the indicated oxygen consumption rate components. **(F)** Acidification measurements were acquired using the Seahorse XF24 Bioanalyzer set to measure the extracellular acidification rate. ^∗^*p*-value < 0.05, five independent replicates, error bars are SD.

The accumulation of mitochondrial ROS suggests that the HAART drugs may be affecting mitochondria. Since mitochondrial dysfunction is thought to contribute to aging and senescence (Lee and Wei, 2012) we evaluated changes in mitochondrial respiration in HAART-treated HAs by using a seahorse bioanalyzer **(Figure 4A)**. ABC–3TC–RTV treatment for 1 week increased both basal and maximal mitochondrial respiration **(Figures 4B,C)**. There was also an increase in ATP-linked respiration **(Figure 4D)**. This HAART drug treatment induced an increase in mitochondrial proton leak, suggesting that it may contribute to the observed increase in mitochondrial respiration **(Figure 4E)**. Measurement of the extracellular acidification rate indicates that HAART drug treatment increases acidification **(Figure 4F)**. Altogether these results suggest that ABC–3TC–RTV cause over-activation of the mitochondria, which may contribute to increased mitochondrial ROS.

**FIGURE 5:**
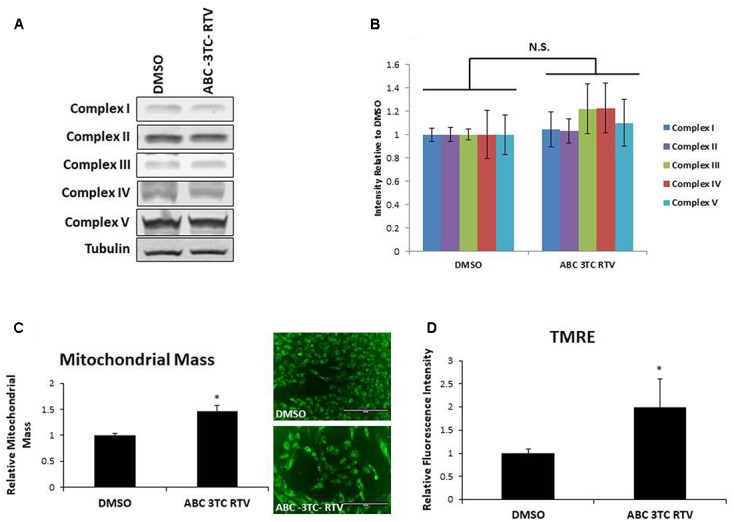
Effect of HAART drugs on mitochondrial mass and mitochondrial membrane potential. Human astrocytes were treated with ABC 10 μM, 3TC 5 μM, and RTV 1 μM for 1 week before assaying. **(A)** Representative Western blot showing protein levels of the mitochondrial electron transport chain complexes. **(B)** Quantification of **A**. **(C)** Mitochondrial mass, cells were incubated with mitotracker for 30 min prior to quantification by flow cytometry as displayed on the left. Right is representative microscopy of fluorescence at 20× before undergoing flow cytometry. **(D)** TMRE, cells were incubated with TMRE for 30 min prior to quantification by flow cytometry. ^∗^*p*-value < 0.05, *n* = 3, error bars are SD.

To determine if the increase in mitochondrial respiration was associated with other changes in the mitochondria, we first examined protein levels of the mitochondrial electron transport chain. As shown in **Figures 5A,B**, HAs treated for 1 week with ABC–3TC–RTV did not induce changes in protein levels of critical components of mitochondrial complexes. However, we observed that mitochondrial mass increased in response to the HAART drug treatment **(Figure 5C)**. Since we were able to detect an increase in mitochondrial respiration and mass, we wanted to determine if this reflects a change in TMRE as a qualitative indicator of mitochondrial membrane potential. Treatment for 1 week with the HAART drugs increased TMRE fluorescence, indicating that the mitochondria may be polarized and activated **(Figure 5D)**.

### HAART Drugs Induce Astrocyte Glycolysis

**FIGURE 6:**
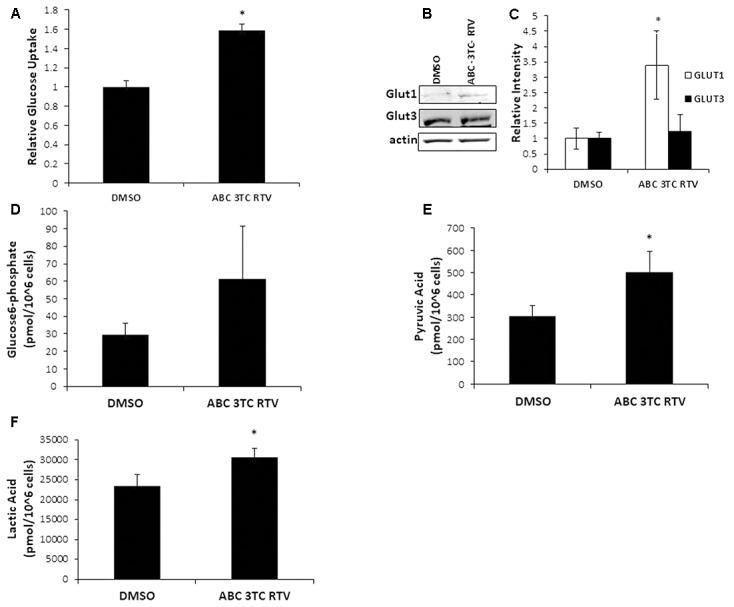
Effect of HAART treatment on astrocyte metabolism. Human astrocytes were treated with ABC 10 μM, 3TC 5 μM, and RTV 1 μM for 1 week before assaying. **(A)** Glucose uptake was measured by flow cytometry after incubation with 2-NDBG for 30 min. **(B)** Western blot illustrating protein levels of Glut1 and Glut3. **(C)** Quantification of B. **(D–F)** Metabolite measurements, metabolites were quantified from HAART-treated cells and untreated by Human Metabolome Technologies Inc. using capillary electrophoresis mass spectrometry. ^∗^*p*-value < 0.05, *n* = 3, error bars are SD.

Highly active antiretroviral therapy drug treatment severely affected astrocyte mitochondrial respiration accompanied by an increased medium acidification, suggesting an altered lactate production as a consequence of enhanced glycolysis. These results are intriguing because senescent fibroblasts were shown to have profound metabolic changes including increased glycolysis (James et al., 2015). We therefore wanted to determine if our astrocytes made senescent from HAART drug treatment have heightened glycolysis. To evaluate directly the effects of HAART on glycolysis we determined changes in glucose and glycolytic intermediates in response to the drugs. First, we examined glucose uptake using a fluorescent glucose analog, 2-NDBG. Highly active antiretroviral therapy drug treatment increased uptake of the glucose analog as measured by flow cytometry **(Figure 6A)**. Glut1, the main glucose transporter in astrocytes was upregulated in response to HAART drug treatment, suggesting that the increase in 2-NDBG uptake could be due to an increase in this transporter. Glut3, which is the main glucose transporter for neurons, is not affected by HAART drug treatment **(Figures 6B,C)**. In order to confirm that there is an increase in glucose metabolism, we examined levels of metabolites associated with glycolysis. Glucose-6-phosphate (G6P), a product produced in the first step of glycolysis trends upward but does not reach statistical significance with HAART drug treatment in HAs **(Figure 6D)**. On the other hand, pyruvate, the last product of glycolysis, does show a statistically significant increase **(Figure 6E)**. Correspondingly, we observed an increase in the production of lactate **(Figure 6F)**, indicating that anaerobic glycolysis is enhanced in response to HAART. This increase in lactate production may explain the increased acidification determined by the Seahorse bioanalyzer **(Figure 4F)**. Overall, these results indicate that HAART drug treatment induces an increase in glucose metabolism in HAs.

## Discussion

The HIV-infected population is growing older and with this increased age, a larger risk for age-associated disease (Niccoli and Partridge, 2012). Neurological issues are particularly troubling because even though HAART has decreased the prevalence of the more severe forms of HAND, the milder forms still remain. We hypothesized that one possible contributor to HAND is the premature induction in astrocytes of cellular senescence in response to HAART drugs. Our study provides evidence for this hypothesis by demonstrating that combinations of HAART drugs are able to induce premature senescence, oxidative stress, mitochondrial dysfunction, and affect glycolysis in HAs. These results are novel since this is the first study to demonstrate HAART drug-induced senescence in a CNS cell type.

We evaluated the impact of widely used NRTIs, NNRTIs, and PIs on primary HAs. These drugs induced various aspects of the senescence program including decreased cellular proliferation, increased SA β-gal and expression of the cell cycle inhibitor p21. While astrocyte senescence has not been explicitly studied in HIV, there is evidence of astrocytes and other glial cells expressing cell cycle inhibitors common to senescence in HIV-infected patients (Jayadev et al., 2007). In concordance, we have previously demonstrated astrocyte senescence in association between AD (Bhat et al., 2012).

Senescent cells are irreversibly growth arrested and their resistance to apoptosis (Childs et al., 2014) allows them to persist in tissues, secreting inflammatory SASP components. The pro-inflammatory microenvironment produced by senescent cells can have major implications *in vivo* since inflammation may contribute to age-related decline in organ function (Freund et al., 2010). Significantly, CNS inflammation has been implicated in neurological disorders such as AD and Parkinson’s disease (Jabbari Azad et al., 2014; Yan et al., 2014). Most importantly, CNS inflammation is found in HIV patients suffering from HAND, even without a productive brain infection (Tavazzi et al., 2014), suggesting that factors other than HIV may be at play. Indeed, our study demonstrates that HAART drug treatment induces the SASP in HAs characterized by the expression of several inflammatory cytokines. Until some of these cytokines are further validated the biological implications of this data are limited. However, IL-6 secretion was demonstrated by ELISA and is known to induce senescence in a paracrine manner (Acosta et al., 2013), potentially allowing for a chain reaction of senescence-inducing-senescence and a chronic inflammatory environment in HIV-infected patients extending beyond the initial insult. Co-culture experiments with HAART drug treated astrocytes and other CNS cell types are thus an important next step to examine this effect *in vitro*. In addition, attenuation of these secretions using anti-inflammatory, SASP modulating, or senescence-delaying drugs could be a potential therapy for HAND.

Dysfunctional mitochondria accumulate with age and can occur both in tissues that contain post-mitotic as well as mitotically active cells (Wallace, 2010). Mitochondrial dysfunction is known to induce cellular senescence both *in vitro* and *in vivo* (Moiseeva et al., 2009), which made it worth looking at how HAART drugs affect the mitochondria in astrocytes. Our HAART drug treated astrocytes display changes in mitochondrial membrane potential, respiration, and mitochondrial ROS production. The production of mitochondrial ROS is particularly interesting as it is thought to be a causal factor in the induction of cellular senescence (Moiseeva et al., 2009). This warrants future studies using antioxidants as a treatment to potentially mitigate HAART drug induced mitochondrial dysfunction and senescence in HAs.

The CNS has extremely high-energy requirements. While only accounting for 2% of human body mass, the CNS is involved in 25% of glucose and 20% of oxygen consumption, indicating that metabolism in the CNS must be tightly controlled. Astrocytes are the key regulators of brain metabolic homeostasis providing nutrition to neurons (Stobart and Anderson, 2013) and changes in astrocyte metabolism can thus have a profound impact on the CNS. Highly active antiretroviral therapy drug treated astrocytes show increased glucose uptake and glycolysis, indicative of a high energy state. While we do not know if this is directly linked to the observed changes in mitochondria, increases in lactate have been observed in patients suffering from mitochondrial myopathies (Kaufmann et al., 2006). The increased utilization of glucose by astrocytes could also potentially impact neurons. While there has been little investigation to link HIV and glycolysis in CNS cells, the effect of HIV on T-cell metabolism has been studied. Glut1 is upregulated on CD4^+^ T-cells of HIV infected patients compared to non-infected controls and these Glut1^+^ cells likewise to our studies also have higher levels of glycolysis (Palmer et al., 2014). When examined *in vitro* not only does HIV infection increase glycolysis of CD4^+^ T-cells, the increased glycolysis also associates with an improved virion production (Hegedus et al., 2014). These results are intriguing because enhanced aerobic glycolysis in the CNS correlates with impaired cognitive function in HIV infected patients (Dickens et al., 2015). Therefore, the increased glycolysis in our HAART drug treated astrocytes may have implications toward HAND. Further significance of our glycolysis results comes from the fact that alterations in glucose have been implicated in AD. Microglia treated with serum from AD patients were found to have increased levels of glycolytic enzymes (Jayasena et al., 2015). In addition, regions of the brain associated with high levels of glycolysis in healthy individuals correlate spatially with Aβ deposits in AD patients (Vlassenko et al., 2010). This suggests that increased levels of glycolysis in the CNS may lead later to Aβ deposits and neurodegeneration.

There are some caveats pertaining to our results. The blood brain barrier hinders penetration of HAART drugs into the CNS, meaning that the doses used to examine the effect of HAART drugs *in vitro* on CNS cells should be lower than plasma levels. However, the relevant parameter to determine physiological levels of HAART drugs in the CNS is a matter of debate. Our long-term (4 weeks) treatments were done using doses based on patient cerebral spinal fluid (CSF). However, HIV patients take these drugs for the rest of their lives and levels could accumulate in cells over a period of years to exceed that of CSF. Levels of ABC in brain homogenates from ABC-treated mice support this (Giri et al., 2008). Therefore, our higher dose 1-week treatments may still be physiological.

The use of HAART drug combinations instead of individual compounds means that the contributions of a specific component cannot be discerned. Since HIV patients take antiretroviral compounds as combinations, we decided to focus the scope of this manuscript accordingly. Interesting future directions include determining if individual antiretroviral drugs or classes are the sole contributors to our astrocyte senescence and dysfunction as well as determining if these adverse effects can be pharmacologically attenuated. In addition, since *in vitro* culturing of fetal astrocytes may not accurately reflect astrocytic function *in vivo*, we want to validate our results by using an *in vivo* model exposing mice to these HAART drugs.

While it is likely that other factors including HIV gene products and drugs of abuse may also contribute to the pathogenesis of HAND *in vivo*, our studies support HAART-induced cellular senescence as a mechanism implied in HAND development. In concordance with our results, clinical trial studies have indicated that drugs with greater CNS penetration resulted in impaired neurocognitive performance, even though HIV was suppressed better (Marra et al., 2009), and that in HIV-infected patients who have preserved immune function, neurocognitive deficits improved after interruption of HAART treatment (Robertson et al., 2010). These results suggest that HAART drugs could still be a major factor in the development of HAND.

## Conclusion

Our data demonstrate that HAs senesce in response to a combination of the HAART drugs ABC–3TC–RTV. This has implications for senescence in the CNS contributing to the neurological problems in patients with HAND. Changes in mitochondria, metabolism, and the secretory profile observed in this study suggest that these are potential targets for therapeutics, which could mitigate HAND.

## Author Contributions

JC conceived and performed the experiments and wrote the manuscript; LD, JW, and FT contributed to the experiments; and CT conceived the experiments and helped write the manuscript.

## Conflict of Interest Statement

The authors declare that the research was conducted in the absence of any commercial or financial relationships that could be construed as a potential conflict of interest.

## Supplementary material

FIGURE S1 Expression of senescence markers in long-term HAART drug treated human astrocytes. Human astrocytes were treated with the following HAART combinations: abacavir (ABC) 3 μM, lamivudine (3TC) 1.9 μM, atazanavir (ATV) 50 nM, and ritonavir (RTV) 100 nM; or tenofovir (TDF) 100 nM, emtricitabine (FTC) 1.2 μM, ATV, and RTV; or TDF, FTC, and efavirenz (EFV) 125 nM for up to 4 weeks in complete astrocyte media. **(A)** Replicative life span curve showing cumulative population doublings as function of days of drug treatments. **(B)** Representative images of 3-week SA β-gal stained cells displayed at 20×. **(C)** SA β-gal activity quantitation. Human astrocytes were stained for SA β-gal 1–3-weeks after HAART treatment. **(D)** Western blot showing protein levels of p16 and p21 after 1 week of treatment. **(E)** IL-6 secretion, conditioned media was collected for 24 h after each treatment. IL-6 was measured by ELISA. ^∗^*p*-value < 0.05, *n* = 3, error bars are SD.

FIGURE S2 Long-term HAART drug treatment induces total and mitochondrial ROS in human astrocytes. Human astrocytes were treated for up to 4 weeks with ABC 3 μM, 3TC 1.9 μM, atazanavir (ATV) 50 nM, and RTV 100 nM. **(A)** Mitochondrial ROS and **(B)** total ROS were measured by 30 min incubation with MitoSox and DCFDA, respectively, before quantification on a flow cytometer. ^∗^*p*-value < 0.05, *n* = 3, error bars are SD.
