# Supplementary figures and images for: Astrocyte Senescence and Metabolic Changes in Response to HIV Antiretroviral Therapy Drugs
Source: Front Aging Neurosci. 2017 Aug 29;9:281. doi: 10.3389/fnagi.2017.00281 (PMC5581874; doi:10.3389/fnagi.2017.00281)

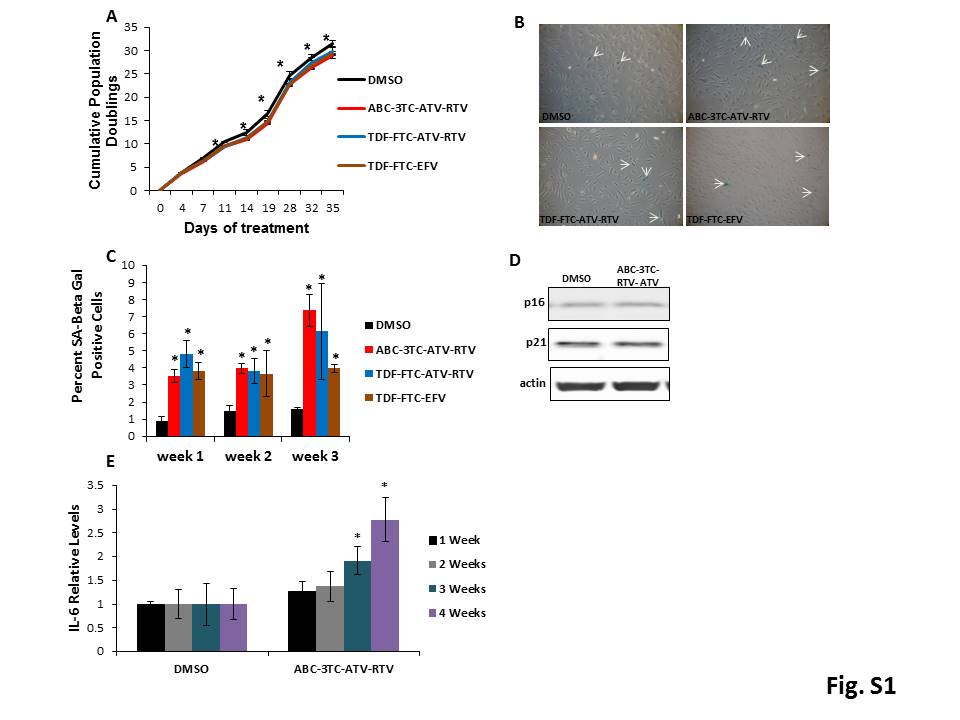

Supplement: FIGURE S1 — Expression of senescence markers in long-term HAART drug treated human astrocytes. Human astrocytes were treated with the following HAART combinations: abacavir (ABC) 3 μM, lamivudine (3TC) 1.9 μM, atazanavir (ATV) 50 nM, and ritonavir (RTV) 100 nM; or tenofovir (TDF) 100 nM, emtricitabine (FTC) 1.2 μM, ATV, and RTV; or TDF, FTC, and efavirenz (EFV) 125 nM for up to 4 weeks in complete astrocyte media. (A) Replicative life span curve showing cumulative population doublings as function of days of drug treatments. (B) Representative images of 3-week SA β-gal stained cells displayed at 20×. (C) SA β-gal activity quantitation. Human astrocytes were stained for SA β-gal 1–3-weeks after HAART treatment. (D) Western blot showing protein levels of p16 and p21 after 1 week of treatment. (E) IL-6 secretion, conditioned media was collected for 24 h after each treatment. IL-6 was measured by ELISA. ∗p-value < 0.05, n = 3, error bars are SD. [file Image_1.JPEG]

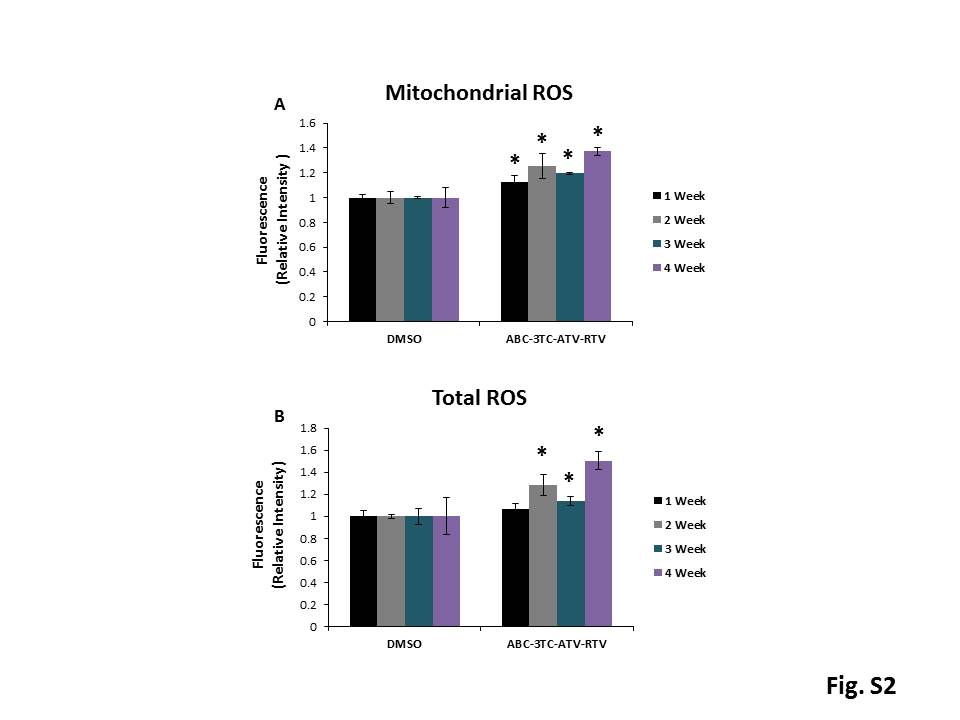

Supplement: FIGURE S2 — Long-term HAART drug treatment induces total and mitochondrial ROS in human astrocytes. Human astrocytes were treated for up to 4 weeks with ABC 3 μM, 3TC 1.9 μM, atazanavir (ATV) 50 nM, and RTV 100 nM. (A) Mitochondrial ROS and (B) total ROS were measured by 30 min incubation with MitoSox and DCFDA, respectively, before quantification on a flow cytometer. ∗p-value < 0.05, n = 3, error bars are SD. [file Image_2.JPEG]
